# Supplementary material for: Modeling glioblastoma heterogeneity as a dynamic network of cell states
Source: Mol Syst Biol. 2021 Sep 16;17(9):e10105. doi: 10.15252/msb.202010105 (PMC8444284; doi:10.15252/msb.202010105)
Supplement: Supplementary file 5 — Source Data for Figure 3 [file MSB-17-e10105-s001.zip › Figure3A_sourcedata/GSEA_3065/nosigdb_state4.GseaPreranked.1622718430994/DOROSZKO_U3013MG_CLUSTER_3.html]

Details for gene set DOROSZKO\_U3013MG\_CLUSTER\_3[GSEA]

|  || Dataset | state4 |
| Phenotype | NoPhenotypeAvailable |
| Upregulated in class | na\_pos |
| GeneSet | DOROSZKO\_U3013MG\_CLUSTER\_3 |
| Enrichment Score (ES) | 0.5338521 |
| Normalized Enrichment Score (NES) | 2.190066 |
| Nominal p-value | 0.0 |
| FDR q-value | 0.0 |
| FWER p-Value | 0.0 |
Table: GSEA Results Summary

  

Fig 1: Enrichment plot: DOROSZKO\_U3013MG\_CLUSTER\_3      
 Profile of the Running ES Score & Positions of GeneSet Members on the Rank Ordered List

  

| PROBE | GENE SYMBOL | GENE\_TITLE | RANK IN GENE LIST | RANK METRIC SCORE | RUNNING ES | CORE ENRICHMENT || 1 | PMP2 |  |  | 3 | 0.727 | 0.0591 | Yes |
| 2 | FABP7 |  |  | 5 | 0.656 | 0.1127 | Yes |
| 3 | CRYAB |  |  | 10 | 0.494 | 0.1525 | Yes |
| 4 | PFN2 |  |  | 46 | 0.303 | 0.1716 | Yes |
| 5 | TSC22D4 |  |  | 49 | 0.298 | 0.1957 | Yes |
| 6 | ID2 |  |  | 56 | 0.292 | 0.2187 | Yes |
| 7 | CTNNA1 |  |  | 71 | 0.274 | 0.2388 | Yes |
| 8 | LGALS3 |  |  | 89 | 0.259 | 0.2573 | Yes |
| 9 | CLU |  |  | 95 | 0.252 | 0.2771 | Yes |
| 10 | TNFRSF21 |  |  | 97 | 0.251 | 0.2975 | Yes |
| 11 | TTYH1 |  |  | 100 | 0.249 | 0.3175 | Yes |
| 12 | CYSTM1 |  |  | 108 | 0.244 | 0.3364 | Yes |
| 13 | PLA2G16 |  |  | 110 | 0.244 | 0.3562 | Yes |
| 14 | VAT1 |  |  | 113 | 0.242 | 0.3757 | Yes |
| 15 | ZNHIT1 |  |  | 120 | 0.238 | 0.3942 | Yes |
| 16 | SNHG18 |  |  | 200 | 0.203 | 0.3981 | Yes |
| 17 | ALKBH7 |  |  | 202 | 0.203 | 0.4145 | Yes |
| 18 | C19orf70 |  |  | 244 | 0.187 | 0.4232 | Yes |
| 19 | EIF4B |  |  | 252 | 0.185 | 0.4372 | Yes |
| 20 | NFE2L2 |  |  | 288 | 0.176 | 0.4460 | Yes |
| 21 | ATP5F1D |  |  | 304 | 0.173 | 0.4577 | Yes |
| 22 | COX14 |  |  | 305 | 0.173 | 0.4719 | Yes |
| 23 | SQSTM1 |  |  | 390 | 0.155 | 0.4710 | Yes |
| 24 | VEGFB |  |  | 414 | 0.149 | 0.4795 | Yes |
| 25 | PHLDA1 |  |  | 464 | 0.142 | 0.4832 | Yes |
| 26 | IFITM3 |  |  | 546 | 0.132 | 0.4809 | Yes |
| 27 | ISCU |  |  | 608 | 0.125 | 0.4812 | Yes |
| 28 | CAMLG |  |  | 639 | 0.121 | 0.4863 | Yes |
| 29 | FAM162A |  |  | 660 | 0.119 | 0.4928 | Yes |
| 30 | VDAC2 |  |  | 795 | 0.104 | 0.4796 | Yes |
| 31 | BAALC |  |  | 816 | 0.103 | 0.4848 | Yes |
| 32 | GLUL |  |  | 819 | 0.102 | 0.4928 | Yes |
| 33 | HIBADH |  |  | 835 | 0.101 | 0.4987 | Yes |
| 34 | BLVRB |  |  | 841 | 0.100 | 0.5061 | Yes |
| 35 | ADI1 |  |  | 863 | 0.099 | 0.5107 | Yes |
| 36 | ISG15 |  |  | 884 | 0.097 | 0.5155 | Yes |
| 37 | NDUFS7 |  |  | 887 | 0.097 | 0.5231 | Yes |
| 38 | BST2 |  |  | 953 | 0.092 | 0.5201 | Yes |
| 39 | MAPT |  |  | 1065 | 0.084 | 0.5089 | Yes |
| 40 | FCGRT |  |  | 1075 | 0.083 | 0.5143 | Yes |
| 41 | ACO2 |  |  | 1077 | 0.083 | 0.5209 | Yes |
| 42 | AL355974.2 |  |  | 1142 | 0.079 | 0.5171 | Yes |
| 43 | NDUFS4 |  |  | 1144 | 0.079 | 0.5234 | Yes |
| 44 | TIMP2 |  |  | 1194 | 0.076 | 0.5217 | Yes |
| 45 | HES4 |  |  | 1197 | 0.076 | 0.5276 | Yes |
| 46 | GDF11 |  |  | 1198 | 0.076 | 0.5339 | Yes |
| 47 | CDH11 |  |  | 1280 | 0.072 | 0.5266 | No |
| 48 | FSTL1 |  |  | 1367 | 0.068 | 0.5182 | No |
| 49 | FRZB |  |  | 1369 | 0.067 | 0.5236 | No |
| 50 | ATP5F1B |  |  | 1379 | 0.067 | 0.5276 | No |
| 51 | GSTK1 |  |  | 1381 | 0.067 | 0.5329 | No |
| 52 | ST20 |  |  | 1432 | 0.065 | 0.5301 | No |
| 53 | HEBP1 |  |  | 1608 | 0.058 | 0.5066 | No |
| 54 | PSAT1 |  |  | 2153 | 0.043 | 0.4219 | No |
| 55 | RAP2B |  |  | 2271 | 0.040 | 0.4062 | No |
| 56 | ISOC2 |  |  | 2278 | 0.040 | 0.4085 | No |
| 57 | ATP6V0E2 |  |  | 2393 | 0.037 | 0.3931 | No |
| 58 | NDUFA12 |  |  | 2515 | 0.034 | 0.3763 | No |
| 59 | MATN2 |  |  | 2545 | 0.034 | 0.3744 | No |
| 60 | P2RX7 |  |  | 2919 | 0.026 | 0.3161 | No |
| 61 | ST8SIA1 |  |  | 2921 | 0.026 | 0.3181 | No |
| 62 | AMD1 |  |  | 3213 | 0.021 | 0.2726 | No |
| 63 | CDK4 |  |  | 3394 | 0.016 | 0.2448 | No |
| 64 | SUCLG1 |  |  | 3497 | 0.012 | 0.2293 | No |
| 65 | NSA2 |  |  | 3503 | 0.012 | 0.2294 | No |
| 66 | MDH1 |  |  | 3601 | 0.009 | 0.2144 | No |
| 67 | LGALS3BP |  |  | 3757 | 0.003 | 0.1896 | No |
| 68 | ADGRG1 |  |  | 3961 | -0.007 | 0.1573 | No |
| 69 | MRPS25 |  |  | 4121 | -0.017 | 0.1329 | No |
| 70 | MIR99AHG |  |  | 4146 | -0.018 | 0.1305 | No |
| 71 | CTSL |  |  | 4153 | -0.019 | 0.1311 | No |
| 72 | RAB27A |  |  | 4217 | -0.023 | 0.1227 | No |
| 73 | PPIC |  |  | 4230 | -0.024 | 0.1228 | No |
| 74 | CTSD |  |  | 4308 | -0.030 | 0.1127 | No |
| 75 | TEX264 |  |  | 4363 | -0.033 | 0.1067 | No |
| 76 | SEMA3E |  |  | 4448 | -0.040 | 0.0963 | No |
| 77 | CYC1 |  |  | 4648 | -0.055 | 0.0686 | No |
| 78 | REV3L |  |  | 4650 | -0.055 | 0.0730 | No |
| 79 | ITGB1BP1 |  |  | 4660 | -0.056 | 0.0761 | No |
| 80 | IFI6 |  |  | 4742 | -0.063 | 0.0682 | No |
| 81 | TKT |  |  | 4829 | -0.070 | 0.0600 | No |
| 82 | SERINC5 |  |  | 5341 | -0.113 | -0.0136 | No |
| 83 | COL18A1 |  |  | 5543 | -0.135 | -0.0350 | No |
| 84 | NAV2 |  |  | 5634 | -0.150 | -0.0374 | No |
| 85 | CANX |  |  | 5755 | -0.173 | -0.0426 | No |
| 86 | MET |  |  | 5815 | -0.187 | -0.0369 | No |
| 87 | CAV1 |  |  | 5842 | -0.193 | -0.0253 | No |
| 88 | IGFBP7 |  |  | 5868 | -0.199 | -0.0131 | No |
| 89 | NRP2 |  |  | 5873 | -0.200 | 0.0026 | No |
| 90 | EMP1 |  |  | 6166 | -0.357 | -0.0154 | No |
| 91 | CD9 |  |  | 6176 | -0.378 | 0.0141 | No |
Table: GSEA details [plain text format]

  

Fig 2: DOROSZKO\_U3013MG\_CLUSTER\_3: Random ES distribution      
 Gene set null distribution of ES for **DOROSZKO\_U3013MG\_CLUSTER\_3**
